# Supplementary material for: The interactome of intact mitochondria by cross-linking mass spectrometry provides evidence for coexisting respiratory supercomplexes
Source: Mol Cell Proteomics. 2017 Dec 8;17(2):216–32. doi: 10.1074/mcp.RA117.000470 (PMC5795388; doi:10.1074/mcp.RA117.000470)
Supplement: Supplemental Data [file supp_RA117.000470_133922_0_supp_23978_fzffwf.zip › spectra_annotation/mito_DR_spectra_annotation/54-1-5-1-5-1.pdf]

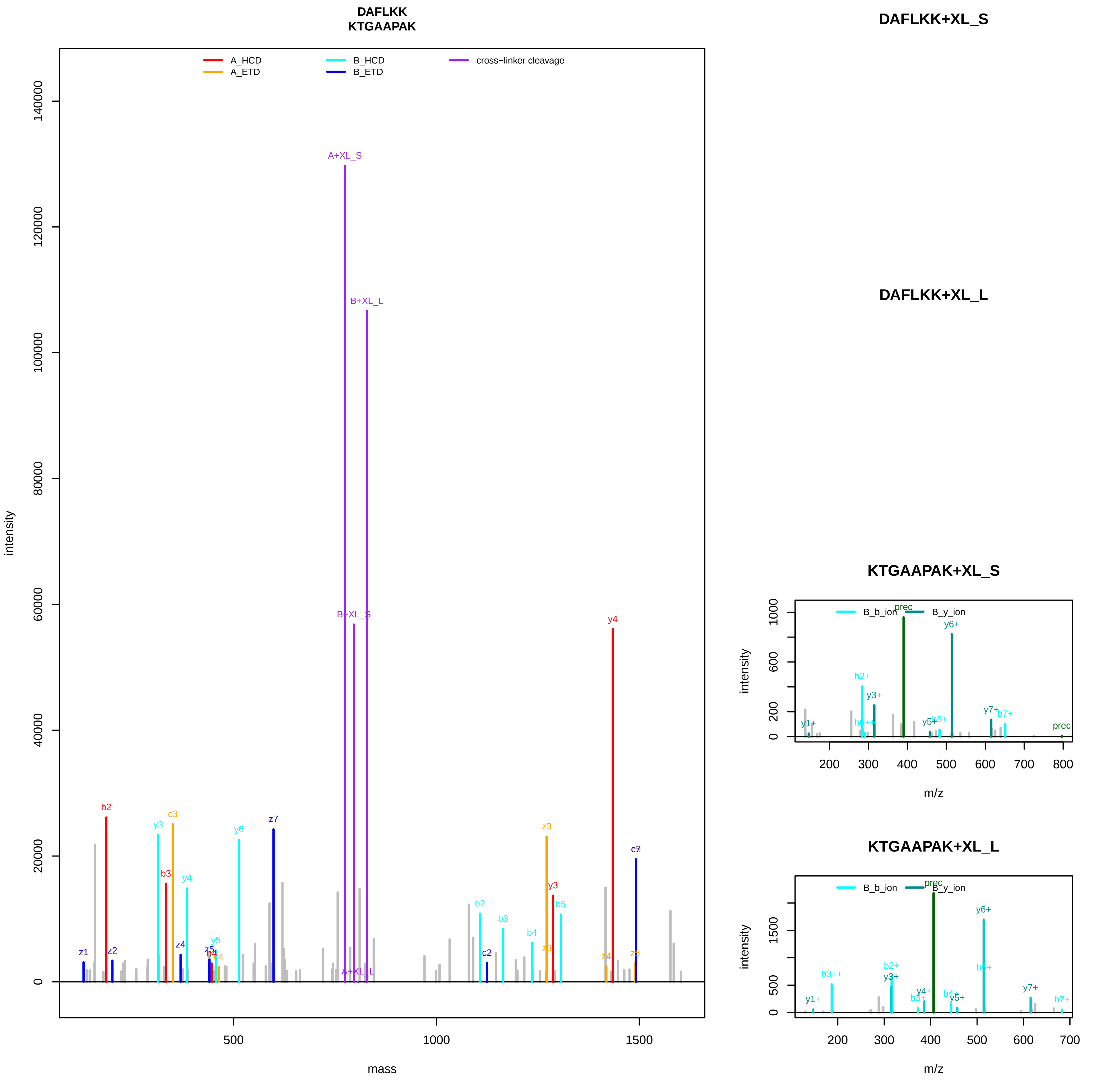
DAFLKK
KTGAAPAK
A_HCD
A_ETD
B_HCD
B_ETD
cross−linker cleavage
A+XL_S
B+XL_L
B+XL_S
A+XL_L
y4
z1
b2
z2
y3
c3
b3
y4
z4
z5
b4
y5
c4
y6
z7
b2
c2
b3
b4
z3
y3
b5
z4
c7
z5
intensity
mass
0
20000
40000
60000
80000
100000
120000
140000
500
1000
1500
DAFLKK+XL_S
DAFLKK+XL_L
KTGAAPAK+XL_S
B_b_ion
B_y_ion
prec
y6+
b2+
y3+
y1+
b6++
y5+
b5+
y7+
b7+
intensity
m/z
KTGAAPAK+XL_L
b3++
b2+
y3+
y4+
b3+
b4+
y5+
b5+
y7+
b7+
